# Physics‐Guided Descriptors Enable Data‐Efficient Prediction of Battery Coulombic Efficiency

**DOI:** 10.1002/advs.76510

**Published:** 2026-09-08

**Authors:** Qintao Sun, Xuewei Gu, Yulin Jie, Hao Yang, Shuhong Jiao, Chao Tang, Yangping Sheng, Yuhao Lu, Ruiguo Cao, Tao Cheng

**Affiliations:** ^1^ Institute of Functional Nano & Soft Materials (FUNSOM) Jiangsu Key Laboratory For Carbon‐Based Functional Materials & Devices Joint International Research Laboratory of Carbon‐Based Functional Materials and Devices P. R. China of Functional Nano & Soft Materials (FUNSOM) Soochow University Suzhou Jiangsu China; ^2^ Hefei National Laboratory For Physical Science At Microscale CAS Key Laboratory of Materials for Energy Conversion Department of Materials Science and Engineering University of Science and Technology of China Hefei Anhui China; ^3^ Ningde Amperex Technology Limited Key Laboratory of Consumer Lithium‐Ion Battery in Fujian Ningde China; ^4^ Jiangsu Key Laboratory of Advanced Negative Carbon Technologies Soochow University Suzhou Jiangsu People's Republic of China

**Keywords:** artificial intelligence, interpretable learning, lithium metal battery, multiscale simulation

## Abstract

Lithium metal batteries promise energy densities beyond 500 Wh kg^−1^; but their practical deployment remains limited by low Coulombic efficiency and uncontrolled electrolyte‐interface reactions. Here, we show that physics‐guided machine learning can identify the molecular origin of Coulombic efficiency (CE) from small experimental datasets by embedding 3D electrolyte structures into data‐driven descriptors. Among the descriptors examined, the physics‐derived solvent‐surrounding‐Li^+^ descriptor (Li^SSL^) enables accurate CE prediction, achieving a test‐set R^2^ of 91.15%. Explainable machine learning further reveals Li^SSL^ as the dominant factor governing model performance, indicating that high‐efficiency lithium deposition requires suppression of direct Li^+–^solvent interactions. This insight establishes a molecular design principle for electrolytes: weakening solvent participation in the primary Li^+^ solvation environment promotes higher CE. Our work provides a physics‐informed, data‐driven framework for accelerating electrolyte discovery toward high‐energy lithium metal batteries.

## Graphical abstract

This work integrates multiscale simulations with data‐driven approaches to predict Coulombic efficiency (CE). Multiscale simulations of battery systems are performed to extract Physics‐Guided descriptors and construct a dataset. Machine learning models trained on this dataset are then subjected to interpretable analysis to identify the most influential descriptors, thereby guiding the rational design of high‐CE electrolytes.

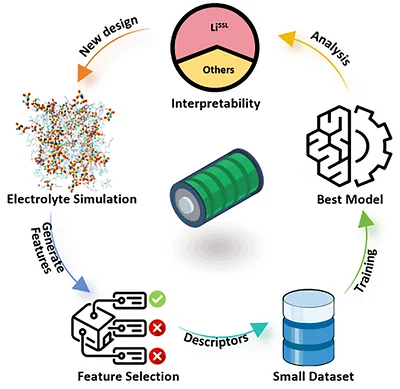

## 1 Introduction

Meeting the escalating global demand for sustainable energy storage requires the development of high‐energy‐density batteries, a pivotal technology for revolutionizing consumer electronics, grid storage, electric vehicles, and emerging low‐altitude airspaces (such as drones, electric vertical take‐off and landing aircraft, and helicopters) [1, 2, 3, 4]. Among various batteries, lithium metal batteries (LMBs), regarded as the holy grail of anode technology, possess unparalleled advantages, such as a high specific capacity of 3860 mAh g^−1^, a low electrochemical potential of 3.04 V compared to that of standard hydrogen electrodes (SHE), and compatibility with diverse cathode materials beyond transition metal oxides, including sulfur and oxygen [5, 6, 7, 8, 9]. The CE is a metric for assessing the cycle stability of LMBs, as it quantifies the efficiency of lithium (Li) transfer during charge–discharge cycles [10, 11, 12]. For LMBs to reach commercial viability at 500 Wh kg^−1^, the CE must exceed 99.95% for 1000 cycles [10]. Unfortunately, LMBs have not yet met these requirements due to challenges such as uncontrollable dendrite growth, parasitic reactions at the solid electrolyte interphase (SEI), and volumetric changes during cycling, which can lead to capacity fade and potential safety hazards [13, 14, 15, 16].

State‐of‐the‐art experimental strategies have been employed to develop long‐term stable LMBs, with significant progress made, particularly through electrolyte engineering, such as the use of high‐concentration electrolytes (HCE), localized high‐concentration electrolytes (LHCE), liquefied gas electrolytes, high‐entropy liquid electrolytes, etc [12, 17, 18, 19]. Recently, a compact ion‐pair aggregate (CIPA) electrolyte that enables high‐performance Li metal pouch cells under lean electrolyte conditions has been reported to achieve long‐life 500 Wh kg^−1^ lithium metal pouch cells [20]. Nevertheless, the commercialization of LMBs remains challenging, considering the historical difficulty of finding a suitable electrolyte for graphite—which is now widely used as an anode in lithium‐ion batteries (LIBs)—after exhaustive research identifying ethylene carbonate (EC) as the skeleton solvent for modern LIBs [21]. Finding an ideal electrolyte for LMBs is even more challenging due to the significantly greater reactivity of lithium metal compared to graphite, which imposes greater constraints, making this task seemingly impossible [22]. The large design space of electrolytes offers hope for finding a suitable (or magical) electrolyte but also presents a significant challenge, as it is impractical and too inefficient to test all possibilities experimentally [23]. Currently, the hope rests in utilizing new tools, especially machine learning (ML) methods, to aid in trial‐and‐error experiments and accelerate the development of electrolytes [24, 25].

Indeed, ML methods have been widely used in battery research and have proven to be highly effective, particularly with rapid advancements in deep learning (DL) [26, 27, 28]. Successful applications in many fields include formula optimization [29], advanced material discovery [30], and battery health monitoring [31]. Recent studies have shown that physics‐guided modeling, signal‐derived feature extraction, and data augmentation can improve battery health prediction. For example, Liu, Yao, Wang, and co‐workers combined RCM HCRE‐based health‐feature extraction with SEI‐constrained BatteryPINN, achieving RMSE and MAPE values below 1% across three datasets [32]. Liu, Liao, Yao, and co‐workers further developed an MGME‐based SOH estimation method with pseudo‐label‐assisted data augmentation, achieving RMSE values of 0.53%, 0.39%, and 1.29% on three datasets [33]. These studies highlight the value of physically informed features and data‐enhancement strategies for battery‐related prediction tasks. Meanwhile, Kim et al. developed ML models using electrolyte composition (hard to confirm the details) to identify key features for a high CE, finding that a lower solvent oxygen content is crucial, and designed fluorine‐free electrolytes achieving a 99.70% CE [34]. Although such a prototype has shown success, this end‐to‐end ML model could achieve greater success if its prediction accuracy and transferability can be improved [26, 35]. Although it has been demonstrated that ML, when combined with a large dataset, is effective for accurate predictions, accurately predicting CE remains challenging due to the small dataset, which is a significant bottleneck in battery research [31, 34, 36]. According to Approximately Correct (PAC) learning theory, to reach 95% confidence with test errors no more than 10%, if an ML model has 10 descriptors, it requires more than 8,500 data points. This number significantly increases to ∼16 000 when the number of descriptors increases to 20. Thus, it is not practical to expect experiments to grow so quickly to meet such requirements, but exploring methods such as feature engineering is a more practical approach for improving the accuracy of ML predictions [37].

Features are critically important for ML, especially when the data size is small [38]. Achieving high accuracy in an ML model with many descriptors (features) and a small data size can seem counterintuitive. Nevertheless, if the descriptors are highly informative and relevant to the target variable, even a small amount of data can be sufficient to capture the underlying relationship [39]. Effective feature selection and dimensionality reduction techniques can help mitigate the risks of overfitting and enhance ML performance [40]. Indeed, feature engineering can transform raw data into meaningful representations, enabling high accuracy even with limited data. As an emerging research area, the prediction of CE for given electrolytes is not yet accurate enough, a gap we are aiming to fill in this work.

The main objective of this study was to develop an accurate ML model that can accurately predict CE for high‐energy‐density LMBs. Inspired by the work of Kim et al. [34], we aim to utilize formula‐based descriptors to predict the CE. Additionally, we propose incorporating descriptors that contain 3D structural information on electrolytes and their chemical and physical properties. We believe that this approach could significantly enhance the prediction accuracy of ML methods for small datasets. To achieve this goal, we performed ab initio calculations to determine the structural and electronic properties of the specified electrolyte components for physical descriptors. These data can be directly utilized as descriptors or employed to derive force fields. At a higher level, molecular dynamics (MD) simulations were conducted to obtain critical information about the electrolyte's 3D atomic structure and properties, which can also serve as descriptors. Following feature selection and refinement, we developed various ML learning methods to enhance the prediction accuracy in both classification and regression tasks. Our approach, derived ab initio and eliminating the need for prior experimental data, promises to bridge existing gaps and set a new standard for accurately predicting CE.

**FIGURE 1:**
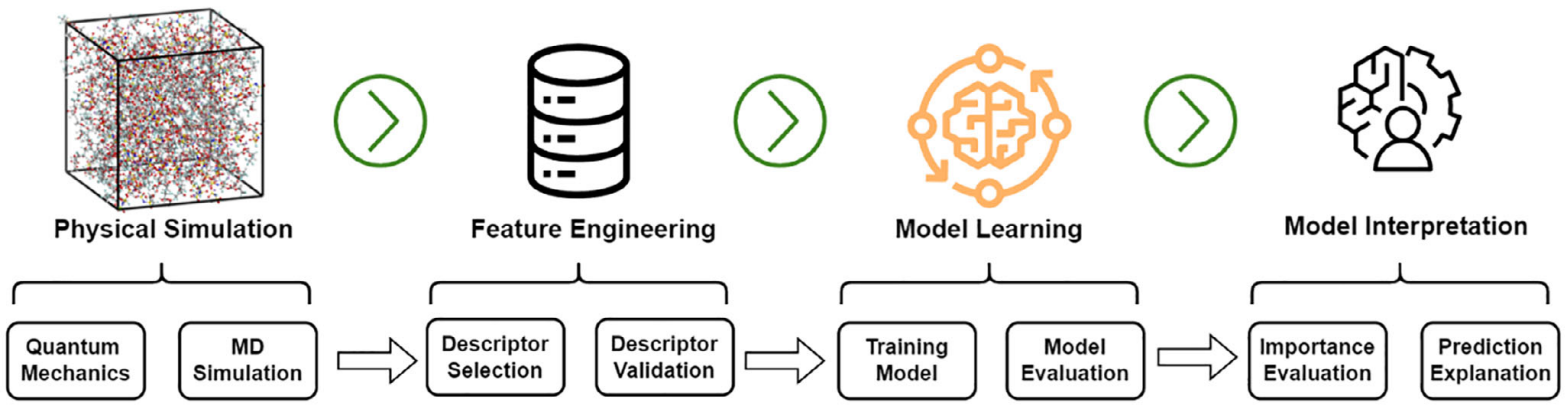
Workflow of machine learning to predict CE. From feature extraction and screening from battery simulations to ML model training and analysis.

To construct an accurate ML model for predicting CE, we designed a workflow (Figure 1) that integrates both multiscale simulation and data‐driven approaches. Initially, the electrolyte structures are optimized using DFT calculations, followed by cMD simulations to capture their dynamic properties. For each electrolyte sample, the complete descriptor‐generation workflow—including molecular‐level calculations, cMD simulations, structural analysis, and descriptor extraction—requires approximately 5 h on a single CPU node equipped with an Intel Xeon Gold 6126 processor. Owing to its high level of automation and parallelizability, this workflow can substantially reduce manual labor and enable cost‐effective, scalable high‐throughput screening. According to the dataset [38, 41], we conducted feature extraction and engineering to extract relevant descriptors, which are known to be crucial for accurate prediction. Subsequent to dataset preparation, the model training phase ensues, where algorithms are not only trained but also rigorously evaluated for their predictive power. Finally, upon achieving a well‐calibrated model, a comprehensive analysis is conducted to understand feature importance, which is then employed for making accurate predictions in CE.

## 2 Results and Discussion

Our ML model, aimed at predicting CE, employs a carefully selected set of 15 descriptors categorized into four groups. These descriptors cover formula‐related parameters (*x_C_, x_H_, x_O_, x_F_, c*), electrolyte properties (ρ, ∆S_mix_, η, ε, *D_Li_*, *D_Anion_*), molecular properties (*E_vdW_*, *E_Coulomb_*, ∆*E*), and solvation structure (Li^SSL^), as Table 1 shows. The inclusion of these four groups allows for comprehensive modeling of CE, capturing various aspects relevant to the property in question. Notably, unlike previously reported ML models [34], our approach incorporates electrolyte 3D descriptors such as solvation structure, enhancing the model's predictive capacity. Having developed an ML model with a diverse range of descriptors, we then assessed the importance of these descriptors through Pearson correlation analysis.

**TABLE 1:** List of variables and the explanation, unit, and data sources.

Type^a^	Descriptor	Explanation	Unit	Source
*a*	*x*_C_	Mole ratio of C	%	Exp., cMD
	*x*_H_	Mole ratio of H	%	Exp., cMD
	*x*_O_	Mole ratio of O	%	Exp., cMD
	*x*_F_	Mole ratio of F	%	Exp., cMD
	*c*	Salt Concentration	mol/L	Exp., cMD
*b*	ρ	Density	g/cm^−3^	Exp., cMD
	Δ*S*_mix_	Entropy of mixing	J·mol^−1^·K^−1^	Exp., cMD
	η	Viscosity	mPa·s	Exp., cMD
	ε	Epsilon	—	Exp., cMD
	*D*_Li_	Li Diffusion coefficient	cm^2^/s	Exp., cMD
	*D*_Anion_	Anion Diffusion coefficient	cm^2^/s	Exp., cMD
*c*	*E*_vdW_	van der Waals interaction	kJ/mol	cMD
	*E*_Coulomb_	Coulombic interaction	kJ/mol	cMD
	*∆E*	HOMO‐LUMO Gap	eV	DFT
*d*	Li^SSL^	Solvent Surrounding Li^+^	%	cMD

^a^ Type *a* means formula‐related descriptors, *b* means descriptors of electrolyte properties, *c* means descriptors of molecular properties, and *d* means descriptors of solvatizon structure.

**FIGURE 2:**
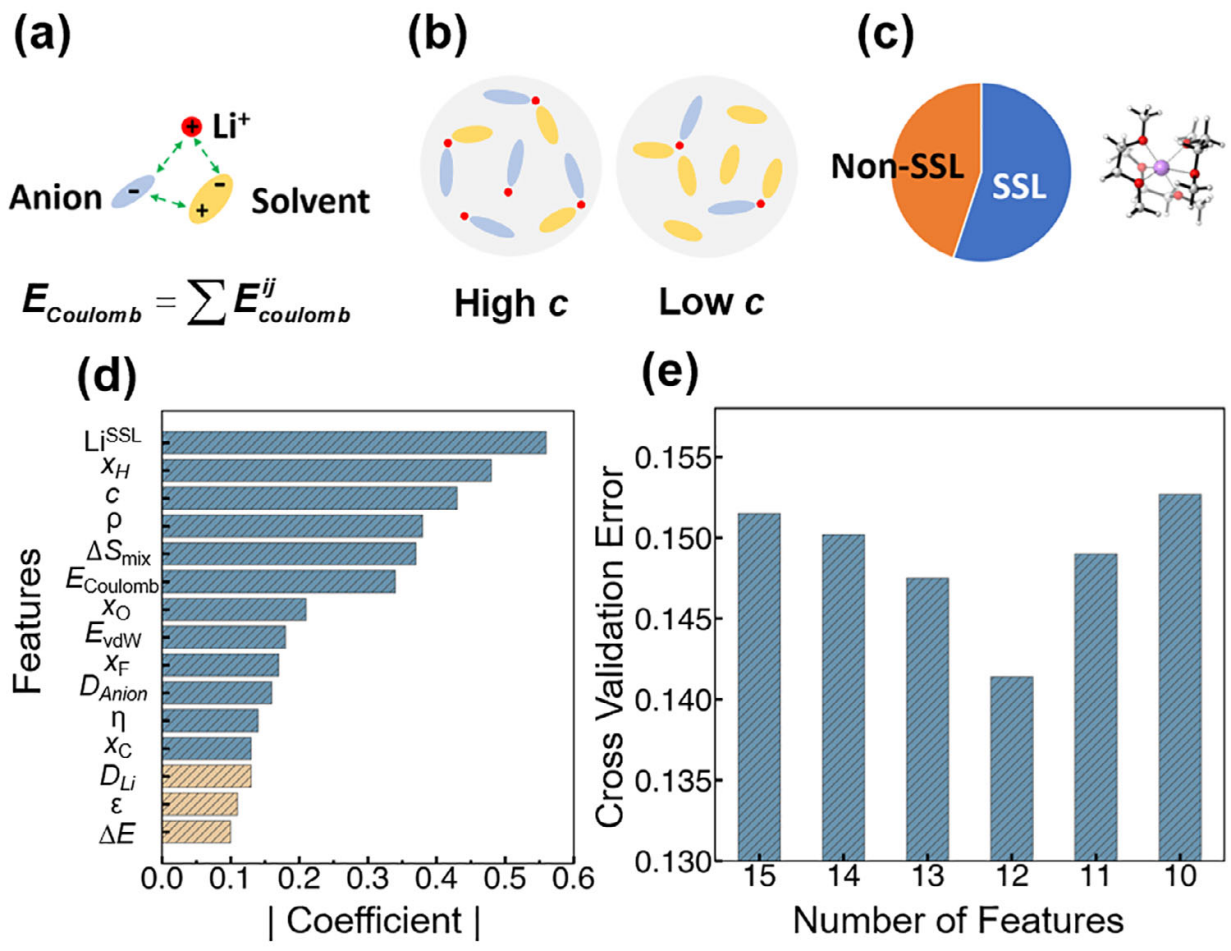
Descriptor construction and feature‐selection analysis. (a) Schematic illustration of the Coulomb interaction energy calculation among Li^+^, anions, and solvent molecules. (b) Schematic comparison of high‐concentration and low‐concentration electrolyte structures. (c) Proportion of Li^+^ solvation structures classified as SSL and non‐SSL, where SSL denotes solvation structures containing Li^SSL^. (d) Absolute Pearson correlation coefficients between candidate descriptors and the target label, *l*‐CE. (e) Cross‐validation error as a function of the number of selected high‐correlation features, showing that the lowest validation error is achieved using the top 12 features.

Pearson correlation analysis revealed the significance of specific descriptors, with Li^SSL^ showing a particularly high correlation with *l*‐CE. As shown in Figure 2d and Figure S4, the degree of correlation is visually indicated by colors: blue indicates positive correlations, and red indicates negative correlations. Li^SSL^ has a correlation of 0.56 with *l*‐CE, while c and ∆*S*_mix_ exhibit negative correlations of −0.43 and −0.37, respectively. The high correlation between Li^SSL^ and the label (*l*‐CE) validates our initial assumption and confirms its importance as a predictive descriptor in the model. This is the first instance where Li^SSL^ has been employed as a descriptor, adding a novel aspect to our study. The Pearson correlation analysis not only confirmed the significance of the selected descriptors but also facilitated the removal of redundant descriptors, as discussed below.

Further Pearson correlation analysis enabled the elimination of descriptors with low correlation with the label, thereby simplifying the model. To select features that are suitable for both linear and non‐linear models, we combined the error of a non‐linear model SVM (with default hyperparameters) to filter out features. Specifically, we observed the error of the non‐linear model by deleting one feature at a time, based on the absolute value of the Pearson correlation coefficient, from lowest to highest order in Figure 2d. As shown in Figure 2e, when keeping only the first 12 features that are correlated with the label, the error of the non‐linear model is minimized. Therefore, we performed feature dimensionality reduction using this method. While this step improved model simplicity, the limitation remains that the number of descriptors is still extensive. Based on PAC learning theory [38], an ML model with 12 descriptors requires 10028 data points to train a well‐performing model. Notably, our confidence in the model's predictive accuracy is contingent because of the highly correlated descriptors selected, such as Li^SSL^. Through Pearson correlation analysis, we not only validated the importance of selected descriptors but also refined the feature set by removing highly intercorrelated descriptors.

**FIGURE 3:**
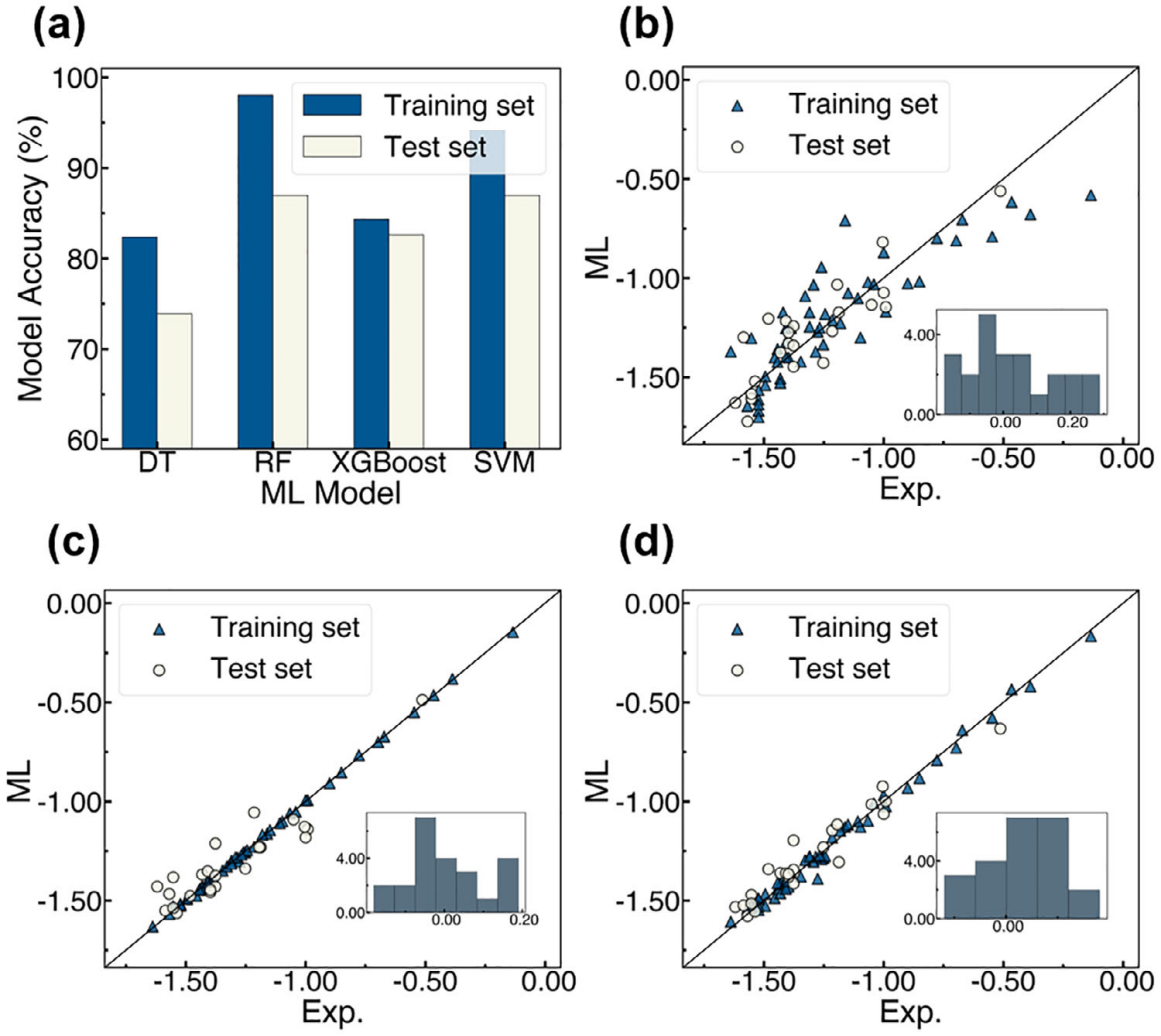
Model performance for classification and regression tasks. (a) Classification accuracy of DT, RF, XGBoost, and SVM models on the training and test sets. Scatter plots of experimental versus predicted *l*‐CE values obtained using the (b) LASSO, (c) XGBoost, and (d) SVM regression models. Insets show the residual distributions, defined as predicted minus experimental *l*‐CE values, for the test set.

To initiate our investigation, we constructed an ML classification model utilizing the selected 12 descriptors, and the label for classification was based on the CE value (Table S3). If the CE is greater than 97%, the label is set to ‘1’; if the CE is between 90% and 97%, the label is set to ‘2’; and if the CE is less than 90%, the label is set to ‘3’. Five ML algorithms are employed: decision tree (DT), random forest (RF), XGBoost, artificial neural networks (ANN), and support vector machine (SVM). DT, RF, and XGBoost were chosen for their excellent interpretability, resilience to outliers, and capacity to capture nonlinear relationships; ANN was utilized for its powerful fitting ability, while SVM was utilized for its robustness in high‐dimensional spaces and flexibility through the kernel trick. Comparative analysis (Table 2 and Figure 3a) revealed that the RF and SVM models outperformed the DT model with an accuracy of 86.96%, while the overfit degree of the SVM model was less than that of the RF model. Additionally, for a comprehensive evaluation of our model, the F1‐score and recall are also applied. The results are listed in Tables S4 and S5, where the recall results are almost consistent with the accuracy results, and the F1‐score results can still exceed 88% in the SVM model. The above results indicate the high predictive capability of the classification model. It should be noted that the classification model is designed to categorize electrolytes into three discrete CE levels—high, medium, and low—rather than to provide quantitative predictions of CE values. With a satisfactory classification model in place, we then developed a regression model for a more precise prediction of CE.

**TABLE 2:** Accuracy of classification models and R^2^ of regression models.

ML Model	Classification		Regression	
	Training (Accuracy)	Test (Accuracy)	Training (R^2^)	Test (R^2^)
LASSO	—	—	0.7593	0.7345
DT	0.8235	0.7391	0.6777	0.5495
RF	0.9804	0.8696	0.6684	0.5623
XGBoost	0.8431	0.8261	0.9995	0.8467
ANN	0.9231	0.8182	0.8848	0.8372
SVM	0.9412	0.8696	0.9904	0.9115

The next step involved the development of an ML regression model to predict CE. In addition to the previously mentioned three algorithms, we considered six models listed in Table 2 to predict the CE value. LASSO was valued for its feature selection capabilities and regularization, XGBoost for its high predictive accuracy and scalability, and ANN for its ability to model complex, nonlinear functions. The R^2^ values of the models are listed in Table 2, and additional model evaluation metrics are listed in Table S7 (MAE) and Table S8 (RMSE). Among these, SVM exhibited the highest performance, with an R^2^ value of 91.15% in the test set, which was supported by data scattering and frequency distribution analysis (Figure 3 and Table 2). Despite the minor difference in R^2^ between the training and test sets, concern arises about the potential overfitting of the model due to the high R^2^. We employed two strategies to examine the degree of overfitting: (1) cross‐validation and (2) prediction on unknown data. Cross‐validation is widely used for assessing overfitting in models [38, 42, 43]. Here, we utilized 5 fold cross‐validation based on the dataset split ratio, and the evaluation results are shown in Table S9. Although the accuracy of most models slightly decreased, the ANN model showed a slight improvement. The decrease in accuracy for most models is not substantial. For example, the best‐performing SVM model maintained an R^2^ of 85.21% in cross‐validation, indicating strong performance. To further assess our model's predictive ability, we extracted data for 12 electrolytes from other studies [34, 44, 45, 46, 47, 48] as our validation set. We conducted multiscale simulations and feature extraction following the same procedure as in Figure 1. Subsequently, the well‐trained SVM model was used for prediction, and the results are presented in Figure S5. The R^2^ on the validation set approached 86.74%, which is comparable to the test set's R^2^ (91.15%) and very close to the cross‐validation results. This further demonstrates the robust predictive capability of our model. To further verify the importance of physics‐guided descriptors, we trained a control model using only formula‐based descriptors, corresponding to Type *a* features in Table 1. The best‐performing model on this formula‐only dataset achieved a test‐set R^2^ of only 0.7119, substantially lower than that obtained using the full descriptor set incorporating 3D structural information (Table S10 and Figure S6). This ablation comparison confirms that cMD/DFT‐derived descriptors and Li^SSL^ provide essential physical information beyond composition alone, thereby improving prediction accuracy, especially for small datasets. Here, physics‐guided descriptors enable data‐driven prediction of Coulombic efficiency, although the framework is not a strictly physics‐constrained machine‐learning model. While these models offer accurate predictions, they fall short of providing insights into the underlying physics as black‐box models. Consequently, to address the question of model interpretability, we conducted SHAP analysis.

**FIGURE 4:**
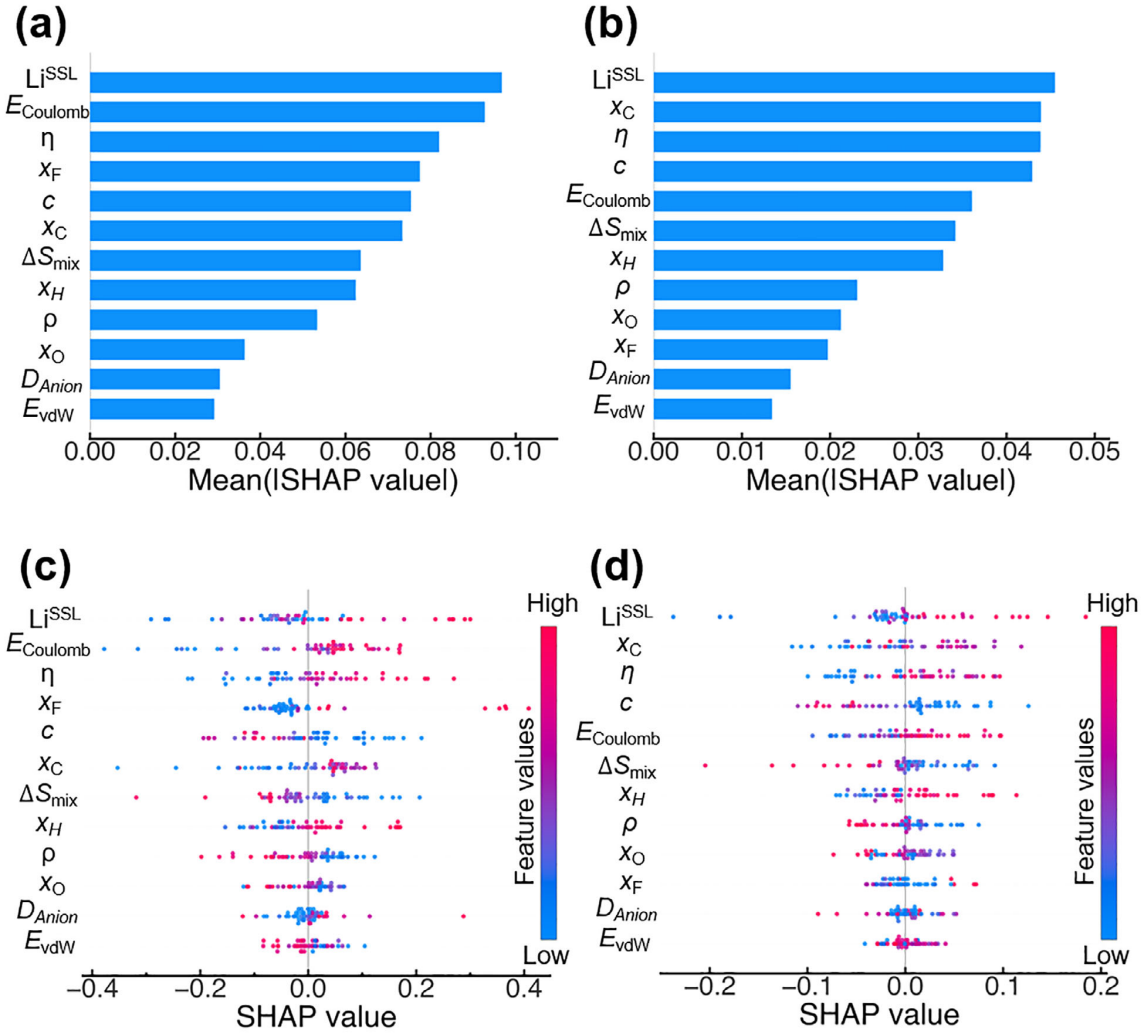
SHAP interpretation of SVM model predictions. Mean absolute SHAP values showing the feature importance of the SVM models for (a) classification and (b) regression tasks. SHAP summary plots showing the feature contributions to the SVM predictions for (c) classification and (d) regression tasks.

To gain further interpretability from our machine learning models, we employed Shapley Additive Explanations (SHAP) analysis. SHAP offers a unified framework for interpreting model outputs, providing both local interpretability and global interpretability across various ML algorithms. A larger mean absolute SHAP value denotes a greater influence of a feature on the prediction outcome, and the colors represent how each feature contributes to the prediction magnitude, with red indicating a positive correlation (larger feature values in high SHAP areas) and blue indicating a negative correlation (larger feature values in low SHAP areas). Our findings confirmed that Li^SSL^ plays a pivotal role in both the classification and regression models, as evidenced in Figure 4a,b. The positive correlation between Li^SSL^ and *l*‐CE observed in Figure 4c,d is consistent with the Pearson correlation analysis (r = +0.56). Specifically, reducing the amount of pure solvent associated with Li^+^ improved the CE. This mechanistic interpretation is consistent with previous studies on electrolyte solvation and interphase chemistry. Li^SSL^ quantifies the fraction of Li^+^ ions involved in solvent‐dominated solvation structures, where solvent molecules occupy most coordination sites in the primary Li^+^ solvation shell with limited anion participation [49]. Such solvent‐dominated structures are generally unfavorable for Li‐metal reversibility for two main reasons. First, Li^+^ must overcome a relatively high desolvation energy barrier by removing strongly coordinated solvent molecules before electrochemical reduction, which can slow interfacial charge‐transfer kinetics and increase polarization [49]. Second, preferential solvent decomposition tends to generate an organic‐rich SEI, which usually exhibits limited ionic conductivity and insufficient stability for regulating repeated Li plating/stripping [50]. In contrast, a lower Li^SSL^ indicates stronger anion participation in the Li^+^ solvation sheath, corresponding to an anion‐involved or anion‐dominated solvation environment. This solvation configuration can facilitate Li^+^ desolvation and promote preferential anion‐derived interphase chemistry at the electrode surface, thereby forming an inorganic‐rich SEI containing species such as LiF and Li_2_O. Such an SEI is generally more mechanically robust and ionically conductive, which benefits stable Li plating/stripping and improves CE [49, 50, 51, 52]. Therefore, although SHAP analysis itself does not establish direct causality, the correlation between Li^SSL^ and CE captured by our model is physically interpretable and mechanistically supported by the established relationship among solvation structure, desolvation kinetics, interphase chemistry, and Li‐metal reversibility. A rigorous causal analysis will be pursued in future studies by combining causal inference, targeted electrolyte design, and controlled experimental/simulation validation.

Nevertheless, we observed certain features with low SHAP values, the importance of which remains unclear. Furthermore, the CE range that the model can predict is restricted to within the training set.

To extend the practical applicability of our findings, we employed LASSO to formulate a mathematical equation for predicting CE as shown in Equation (1). This equation was derived within the constraints of the linear model of LASSO, leveraging its proven capabilities in feature selection and regularization, which could limit the ability of the equation to capture potentially complex, nonlinear relationships affecting CE. To address this limitation, future research could consider employing advanced methods such as Sure Independence Screening and Sparsifying Operator (SISSO) [39] for potentially improved predictive performance.

(1) $$\begin{array}{ccc} l-\mathrm{CE}\; & = & -0.6925\times F\left(E_{vdW}\right)-0.0551\times F\left(E_{Coulomb}\right)+0.2441\\ & & \times \;F\left(\eta \right)-0.7639\times F\left(\rho \right)-0.0045\times F\left(D_{Anion}\right)+0.1635\\ & & \times \;F\left(Li^{SSL}\right)-0.1401\times F\left(\Delta S_{mix}\right)+0.2569\times F\left(c\right)\\ & & -\;0.0509\times F\left(x_{C}\right)-0.2394\times F\left(x_{O}\right)+0.7151\\ & & \times \;F\left(x_{F}\right)+0.3646\times F\left(x_{H}\right)-1.2301 \end{array}$$

## 3 Conclusion

To summarize, an accurate ML model has been developed to predict the CE of LMBs. A dataset of 74 battery samples from the experiment was used to train the ML model. We meticulously chose 12 descriptors that not only encompass the traditionally utilized formula information but also incorporate 3D electrolyte details, specifically the Li^SSL^. Various ML models were trained and evaluated on this dataset. The RF and SVM classifier achieved the highest accuracy, exceeding 86.96% on the test set, while the SVM regression model performed well in predicting CE, with an R^2^ of 91.15% and an RMSE of 0.08. Furthermore, the evaluations through both cross‐validation and additional validation sets confirmed the robust predictive capability of our model. SHAP analysis revealed that Li^SSL^ is the most influential feature for accurately predicting the CE. Based on the ML model, we propose an experiment in which an electrolyte that minimizes Li^+^ association solely with a solvent is chosen to enhance battery performance. Furthermore, we derived a mathematical equation based on LASSO. This equation serves as a transferable prediction model for CE, enabling us to make accurate predictions beyond the training set. To address the current limitations of our model, we intend to exclude formula‐related parameters from the feature set in future iterations. Additionally, we plan to implement a regression analysis using SISSO to capture nonlinear relationships within the equation.

## 4 Experimental Section

### 4.1 Density Functional Theory Calculations

Density functional theory (DFT) calculations were performed using Gaussian 16 [53]. The Perdew‐Burke‐Ernzerhof (PBE) functional [54], 6–31+G* basis set [55], and D3‐BJ [56] were used as the default choices for the calculations. The PBE functional does not require empirical scaling of its Hessian matrix, making it a suitable option for matching experimental frequencies. Atomic charges were determined via Charge Model 5 (CM5) [57], based on Hirschfeld partitioning [58, 59, 60] adjusted at a ratio of 1.2 for neutral molecules to account for condensed‐phase polarization for the force field with a point charge model. Relaxed dihedral scans were performed to determine the corresponding dihedral energy profiles. Consistent with previous research, the energy gap (*∆E*) between the highest occupied molecular orbital (HOMO) and lowest unoccupied molecular orbital (LUMO) was calculated at the B3LYP/6‐311+G** level.

### 4.2 Classical Molecular Dynamics Simulations

The GROMACS 2020 software package [61] was used for classical molecular dynamics (cMD) simulations. To simulate the electrolyte, a simulation box with dimensions of approximately 4 nm was constructed, which contains lithium salts and solvent molecules with ratios according to the experimental formulation. The cMD simulation workflow involves optimization, followed by a 1 ns canonical (NVT) simulation at 300 K using a V‐rescale thermostat, a 10 ns isothermal‐isobaric (NPT) simulation at 300 K and a 1.0 bar pressure using a Nose‒Hoover thermostat. This was then followed by either a 10 ns NPT production simulation or a 10 ns NVT production simulation. To verify whether the NPT reached equilibrium, we plotted the change in system density over time throughout the entire simulation process. Due to the large number of electrolytes, we selected several typical electrolytes, including low‐concentration, high‐concentration, and locally high‐concentration electrolytes. As shown in Figure S3, the majority of the systems reached equilibrium by 1 ns. These cMD simulations provide data regarding the behavior and properties of the individual components present in the electrolyte, such as conductivity and intermolecular interaction ensembles, which were used for equilibration at 300 K and 1.0 bar, followed by the NVT ensemble for further simulation. Properties, including density (ρ), van der Waals (*E_vdW_*), Coulomb interaction (*E_Coulomb_*), viscosity (η), epsilon (ε), and diffusion coefficients (*D*), were derived from cMD simulations (for details, see the supplementary materials).

### 4.3 Machine Learning Models

As shown in Table 1, 15 descriptors are considered, including the mole ratio of C, H, O, and F (*x_C_*, *x*_H_, *x*_O_, *x*_F_) in the electrolytes, salt concentration (*c*), density (ρ), entropy of mixing (∆*S*_mix_), viscosity (η), diffusion coefficient of anions (*D*_Anion_), van der Waals interaction (*E*_vdW_), Coulomb interaction (*E*_Coulomb_), HOMO–LUMO gap (*∆E*), and solvent–surrounding–Li^+^ (Li^SSL^). These descriptors are either from formula, cMDs, or *DFT simulations* the label (*y*) is log (1 – CE), denoted as *l*‐CE, using 74 experimental CE data [62]. These 74 electrolytes are single or mixed solvents composed of 12 solvents and LiFSI. The molecular structures of the solvent molecules are shown in Figure S2. Given the inverse relationship between CE and *l*‐CE, lower values of *l*‐CE are preferable for achieving a high CE [34].

The dataset was randomly divided into a training set comprising 70% of the data and a testing set comprising 30% of the data. To further evaluate the model's performance and assess its overfitting degree, a 5 fold cross‐validation method was employed to validate the model's generalization ability. To evaluate the performance of various ML models, the accuracy, mean absolute error (MAE), root mean square error (RMSE), coefficient of determination (R^2^), F1‐score, and Recall score are utilized as metrics; more details are in Supporting Information.

The ML algorithms used in this work includes Least Absolute Shrinkage and Selection Operator (LASSO) [63], Decision Tree (DT) [64], Random Forest (RF) [65], Extreme Gradient Boosting (XGBoost) [66], Support Vector Machine (SVM) [67] as implemented in Scikit‐learn and Artificial Neural Network (ANN) [68] as implemented in TensorFlow.

To gain a comprehensive understanding of the predictions made by the black‐box ML model, Shapley Additive exPlanations (SHAP) [69] were utilized to interpret and provide an in‐depth understanding of the feature importance of CE.

## Author Contributions

**Qintao Sun**: investigation, writing – original draft, visualization, methodology. **Tao Cheng**: conceptualization, investigation, formal analysis, supervision, funding acquisition, writing – original draft, writing – review and editing. **Yuhao Lu**: supervision, investigation, formal analysis, writing – original draft, conceptualization. **Hao Yang**: investigation, writing – original draft, formal analysis. **Shuhong Jiao**: formal analysis, supervision, writing – original draft. **Ruiguo Cao**: investigation, supervision, writing – original draft, conceptualization. **Chao Tang**: supervision, writing – original draft, investigation. **Yangping Sheng**: investigation, validation, writing – original draft, formal analysis, conceptualization. **Yulin Jie**: investigation, validation, writing – original draft. **Xuewei Gu**: writing – original draft, investigation, validation.

## Conflicts of Interest

The authors declare no conflicts of interest.

## Supporting information




**Supporting File**: advs76510‐sup‐0001‐SuppMat.docx.

## Data Availability

The data that support the findings of this study are available on request from the corresponding author. The data are not publicly available due to privacy or ethical restrictions.
