# Supplementary material for: Physics‐Guided Descriptors Enable Data‐Efficient Prediction of Battery Coulombic Efficiency
Source: Adv Sci (Weinh). 2026 Sep 8;13(57):e76510. doi: 10.1002/advs.76510 (PMC13554369; doi:10.1002/advs.76510)
Supplement: Supplementary file 1 [file ADVS-13-e76510-s001.docx]

Supporting Information

Physics-Guided Descriptors Enable Data-Efficient Prediction of Battery Coulombic Efficiency

Qintao Sun^a^, Xuewei Gu^a^, Yulin Jie^b^, Hao Yang^a^, Shuhong Jiao^b^, Chao Tang^c, d^, Yangping Sheng^c,^*, Yuhao Lu^c,^*, Ruiguo Cao^b,^* and Tao Cheng^a,^^d,^*

^a^ Institute of Functional Nano & Soft Materials (FUNSOM), Jiangsu Key Laboratory for Carbon-Based Functional Materials & Devices, Joint International Research Laboratory of Carbon-Based Functional Materials and Devices, Soochow University, 199 Ren’ai Road, Suzhou, 215123, Jiangsu, P. R. China of Functional Nano & Soft Materials (FUNSOM).

^b^ Hefei National Laboratory for Physical Science at Microscale, CAS Key Laboratory of Materials for Energy Conversion, Department of Materials Science and Engineering, University of Science and Technology of China, Hefei, Anhui, 230026, China.

^c^ Ningde Amperex Technology Limited, Key Laboratory of Consumer Lithium-Ion Battery in Fujian, Ningde 352100, China.

^d^ Jiangsu Key Laboratory of Advanced Negative Carbon Technologies, Soochow University, Suzhou, 215123, Jiangsu, PR China.

E-mail: [shengyp@atlbattery.com, luyuh@atlbattery.com](mailto:shengyp@atlbattery.com,%20luyuh@atlbattery.com), [rgcao@ustc.edu.cn](mailto:rgcao@ustc.edu.cn), [tcheng@suda.edu.cn](mailto:tcheng@suda.edu.cn)

***I. Supporting Text***

1. ***Methods***

***1.1 The computational details of all features.***

1.1.1 *x*_C_, *x*_H_, *x*_O_, *x*_F_ in **Table 1**, which means the mole ratio of C, the mole ratio of F, the mole ratio of O, the mole ratio of H.

The ratio of carbon, hydrogen, oxygen, and fluorine is calculated as follows:

$x_{i} = \frac{i element number}{V_{periodic box} }$ (S1)

where $V_{periodic box}$ is the simulated box size obtained by the NPT ensemble molecular dynamics simulation.

1.1.2 *c* in **Table 1**, which means concentration of electrolytes.

The concentration of electrolytes is calculated by the following equation:

$c = \frac{n_{salt}}{V_{periodic box}}$ (S2)

where $V_{periodic box}$ is the simulated box size obtained by the NPT ensemble molecular dynamics simulation, and $n_{salt}$ is the mole number of salt in the electrolyte.

1.1.3 ρ in **Table 1**, which indicates the density of electrolytes.

The density of the electrolyte systems was calculated with the following equation:

$\rho= \frac{\text{mass}}{V_{periodic box}}$ (S3)

where$V_{periodic box}$ is the simulated box size obtained by the NPT ensemble molecular dynamics simulation.

1.1.4 $\Delta S_{mix}$ in **Table 1**, which indicates the entropy of mixing.

The entropy of mixing is calculated as follows ^[1]^:

$\Delta S_{mix} = -R\sum_{i=1}^{N} c_{i}lnc_{i}$ (S4)

where *R* is the ideal gas constant, *N* is the type of substance, and *c* is the content.

1.1.5 η in **Table 1**, which means simulated viscosity.

The shear viscosity of the liquid was estimated by calculating the training-collecting autocorrelation functions (TCAFs). Transverse currents are calculated using the *k*-vectors (1,0,0) and (2,0,0) in the y- and z-directions, (1,1,0) and (1, -1,0) in the 2 other planes (these vectors are not independent) and (1,1,1) and the 3 other box diagonals (also not independent). For each k-vector, the sine and cosine are used, in combination with the velocity in 2 perpendicular directions. This gives a total of 16×2×2=64 transverse currents. One autocorrelation is calculated fitted for each k-vector, which gives 16 TCAFs. Each TCAF is fitted as^[2]^:

$f(t) = \mathrm{e}^{(-v)(\cosh(wv)+1/w \sinh(wv))}$ (S5)

$v = -t/(2 \tau)$ (S6)

$w = (1-4tau eta/rho k^{2})^{1/2}$ (S7)

to obtain the values of 16$\tau$ and η. The fitting weight decays exponentially with time constant w, namely, exp (-t/w), TCAF and fitting are calculated to 5×w.

1.1.6 *D*_Li_ and *D*_Anion_ in **Table 1**, which means the diffusion coefficient of Li^+^ and anions.

The mean square displacement (MSD) of atoms from a set of initial positions is fitted to the calculated diffusion constant. There is a simple way to calculate the diffusion constant of the particles, which is based on the Einstein relation ^[3]^:

$\lim_{t\to\infty} \left\langle\left\|r_{i}(t)-r_{i}(0)\right\|^{2}\right\rangle_{i\in A} = 6D_{A}t$ (S8)

The diffusion coefficient (*D*_A_) is determined by linear regression of the MSD.

1.1.7 *E*_vdW_ in **Table 1**, which means van der Waals interaction energy, here the energies are divide by $V_{periodic box}$ to normalization.

The van der Waals (vdW) interaction energy is a sum of short-range and long-range interactions, and the short-range interaction energy is *E_LJ(SR)_*, while the long-range part is reflected in the form of dispersion correction, namely, *E_Disper.-corr_*. The vdW interaction energy is calculated as the sum of *E_LJ(SR)_* and *E_Disper.-corr_*_._

The Lennard‒Jones (LJ) 12-6 potential $V_{LJ}$ (*E_LJ(SR_*_)_) between two atoms equals:

$V_{LJ}(r_{ij}) = \frac{c_{ij}^{(12)}}{r_{ij}^{12}} - \frac{c_{ij}^{(6)}}{r_{ij}^{6}}$ (S9)

The parameters $c_{ij}^{(12)}$ and $c_{ij}^{(6)}$ depend on pairs of atom types; consequently, they are taken from a matrix of LJ parameters. We define the lowercase subscripts $i$ and $j$ to denote particles: $\mathbf{r}_{i}$ is the position vector of the $i$ particle, and using this notation: $\mathbf{r}_{ij}$=$\mathbf{r}_{j}$-$\mathbf{r}_{i}$, $\boldsymbol{r}_{ij}$=$\left| \mathbf{r}_{ij} \right|$

To take the long-range interaction into consideration, the long-range contribution of the dispersion interaction to the virial can be derived analytically. In a periodic system, it is not easy to calculate the full potentials, so usually a cutoff is applied, which can be abrupt or smooth. We will call the potential and force with cutoff $V_{c}$. The long-range contribution to the dispersion energy (*E_Disper.-corr_*_._) in a system with *N* particles and particle density $\rho=N/V$ is:

$V_{lr}= \frac{1}{2}N\rho\int_{0}^{\infty} 4\pi r^{2}g(r)(V(r) - V_{c}(r))dr$ (S10)

where$g(r)$ is the radial distribution function.

1.1.8 *E*_Coulomb_ in **Table 1**, which means Coulomb interaction energy, here the energies are divide by $V_{periodic box}$ to normalization.

The Coulomb interaction between two charge particles is given by:

(S11)

where $f = \frac{1}{4\pi\varepsilon_{0}} =$ 138.935458, $\epsilon_{r}$ is the relative dielectric constant, and ***q*** is the quantity of electric charge.

1.1.9 ∆*E* in **Table 1**, which means a gap between the HOMO and LUMO energies.

The gap between the HOMO and LUMO energies in a single solvent is calculated as follows:

$E_{Gap} = E_{LUMO} - E_{HOMO}$ (S12)

The DFT calculations are conducted at the b3lyp/6-311+g** level.

The solvent gap of the HOMO and LUMO energies in electrolytes is calculated as follows:

$\Delta E = \sum_{\text{i} = 1}^{k} \frac{N_{i}}{N_{solvent}}E_{Gap_{i}}$ (S13)

where *k* is the type of solvent, $N_{solvent}$is the number of solvent molecules, and $Gap_{i}$is calculated by equation (S12):

1.1.10 Li^SSL^ in **Table 1**, which means the percentage of solvent surrounding Li^+^.

The percentage of Li^SSL^ is calculated by：

$Li^{SSL} = \frac{N_{Li surrounded by solvent}}{N_{Li}}$ (S14)

The Li^SSL^ are obtained from the classical MD, and the cutoff for the coordination of Li-salt or Li-solvent is 2.7 Å.

***1.2 Details of model evaluation***

$Accuracy = \frac{n_{ap}}{n}$ (S15)

$MAE = \frac{1}{n}\sum_{i=1}^{n} \left|y_{i}-\hat{y}_{i}\right|$ (S16)

$RMSE = \frac{1}{n}\sum_{i=1}^{n} \sqrt{\left(y_{i}- \hat{y}_{i}\right)^{2}}$ (S17)

$R^{2} = 1-\frac{\sum_{i=1}^{n} (y_{i}-\hat{y}_{i})^{2}}{\sum_{i=1}^{n} (y_{i}- \bar{y})^{2}}$ (S18)

where $n_{ap}$ is the number of samples that were accurately predicted, n is the number of samples in the training set or test set, $y_{i}$ is the label value of sample i, $\hat{y}_{i}$ is the predicted value of sample i, and $\bar{y}$ is the average label value.

***II. Supporting Figures***

**Figure S1.** Flowchart of the development of classical force field parameters.

**Figure S2.** The structural diagram of solvent molecules and salt anion composing the electrolyte in the dataset. Color code: oxygen, red; carbon, gray; fluorine, cyan; sulfur, yellow, nitrogen, blue; hydrogen, white.

**Figure S3.** Variation of electrolyte density over time in NPT simulation process. a. 1mol LiFSI/DME; b. 1.5mol LiFSI/DME:HFE(9:1); c. 5.6mol LiFSI/THF; d. 3.5mol LiFSI/FEMC.

**Figure S4.** Pearson correction study of the dataset with 15 features.

**Figure S5.** Scatter plot of predicted values versus experimental values on the validation set by the well-trained SVM model. The validation set data is sourced from the literature ^[4-9]^.

**Figure S6**. **Ablation analysis of model performance using formula-only descriptors.** Model performance obtained using only composition-based descriptors, corresponding to Type *a* features in **Table 1**. (a) Accuracy of the four classification models. Scatter plots of the training values against the predicted values for the prediction of *l*-CE by the (b) LASSO model, (c) XGBoost model, and (d) SVM model. Insets show histograms of residuals (predicted - experimental) for the test set.

***Ⅲ. Supporting Tables***

**Table S1.** The optimized hyperparameters of the classification models. (Note: default values are used for the unmentioned hyperparameters.)

| ML model | Hyperparameters | Value |
| --- | --- | --- |
| Decision Tree | max_depth | 2 |
|  | random_state | 0 |
| Random Forest | n_estimators | 10 |
|  | random_state | 0 |
| XGBoost | n_estimators | 4 |
|  | min_child_weight | 3 |
|  | reg_lambda | 1.128 |
|  | random_state | 0 |
| ANN | learning rate | 0.001 |
|  | hidden layers | 4 |
|  | number of neurons on every layer | (58,1,197,77) |
|  | activation function | relu |
|  | optimizer | adam |
|  | epochs | 200 |
|  | random_seed | 0 |
|  | random state | 35 |
| SVM | kernel | rbf |
|  | C | 8 |
|  | gamma | 0.074 |

**Table S2.** The optimized hyperparameters of regression models. (Note: default values are used for the unmentioned hyperparameters.)

| ML model | Hyperparameters | Value |
| --- | --- | --- |
| LASSO | alpha | 1e-14 |
| Decision Tree | max_depth | 6 |
|  | min_samples_leaf | 9 |
|  | random_state | 0 |
| Random Forest | n_estimators | 41 |
|  | max_depth | 12 |
|  | min_samples_leaf | 6 |
|  | random_state | 0 |
| XGBoost | n_estimators | 100 |
|  | min_child_weight | 8 |
|  | reg_lambda | 1.208 |
|  | random_state | 0 |
| ANN | learning rate | 0.001 |
|  | hidden layers | 4 |
|  | number of neurons on every layer | (58,1,197,77) |
|  | activation function | relu |
|  | optimizer | adam |
|  | epochs | 200 |
|  | random_seed | 0 |
|  | random state | 35 |
| SVM | kernel | rbf |
|  | C | 8 |
|  | gamma | 0.175686 |
|  | epsilon | 0.032054 |

**Table S3.** The classification basis in the classification dataset.

| **CE (%)** | **Class degree** | **Label** | | **N_samples_** |
| --- | --- | --- | --- | --- |
| [97,100] | High | | 1 | 14 |
| [90,97) | Medium | | 2 | 48 |
| [0,90) | Low | | 3 | 12 |

**Table S4.** Evaluation Metrics (F1-score) Value on the Classification Models.

| **ML Model** | **Train (F1-score)** | **Test (F1-score)** |
| --- | --- | --- |
| Decision Tree | 0.8020 | 0.6739 |
| RFR | 0.9799 | 0.8596 |
| XGBoost | 0.8413 | 0.8183 |
| ANN | 0.9249 | 0.8159 |
| SVM | 0.9397 | 0.8804 |

**Table S5.** Evaluation Metrics (Recall Score) Value on the Classification Models.

| **ML Model** | **Train (Recall Score)** | **Test (Recall Score)** |
| --- | --- | --- |
| Decision Tree | 0.8235 | 0.7391 |
| RFR | 0.9804 | 0.8696 |
| XGBoost | 0.8431 | 0.8261 |
| ANN | 0.9231 | 0.8182 |
| SVM | 0.9412 | 0.8696 |

**Table S6.** 5-fold Cross-validation Evaluation for the Classification Model.

| **ML Model** | **Accuracy** | **F1-score** | **Recall score** |
| --- | --- | --- | --- |
| Decision Tree | 0.6895 | 0.6427 | 0.6895 |
| RFR | 0.7705 | 0.7403 | 0.7705 |
| XGBoost | 0.7162 | 0.6849 | 0.7162 |
| ANN | 0.6486 | 0.6033 | 0.6486 |
| SVM | 0.7562 | 0.7556 | 0.7562 |

**Table S7.** Evaluation Metrics (MAE) Value on the Regression Models.

| **ML Model** | **Train (MAE)** | **Test (MAE)** |
| --- | --- | --- |
| LASSO | 0.1229 | 0.1071 |
| Decision Tree | 0.1340 | 0.1510 |
| RFR | 0.1433 | 0.1543 |
| XGBoost | 0.0049 | 0.0777 |
| ANN | 0.0715 | 0.0976 |
| SVM | 0.0287 | 0.0629 |

**Table S8.** Evaluation Metrics (RMSE) Value on the Regression Models.

| **ML Model** | **Train (RMSE)** | **Test (RMSE)** |
| --- | --- | --- |
| LASSO | 0.1629 | 0.1327 |
| Decision Tree | 0.1885 | 0.1729 |
| RFR | 0.1912 | 0.1704 |
| XGBoost | 0.0077 | 0.1008 |
| ANN | 0.1045 | 0.1325 |
| SVM | 0.0326 | 0.0766 |

**Table S9.** 5-fold Cross-validation Evaluation for the Regression Model.

| **ML Model** | **R^2^** | **MAE** | **RMSE** |
| --- | --- | --- | --- |
| LASSO | 0.5701 | 0.1417 | 0.1902 |
| Decision Tree | 0.2266 | 0.2100 | 0.2648 |
| RFR | 0.4401 | 0.1824 | 0.2287 |
| XGBoost | 0.7217 | 0.1232 | 0.1674 |
| ANN | 0.8833 | 0.0714 | 0.0893 |
| SVM | 0.8521 | 0.0818 | 0.1136 |

**Table S10.** **Ablation analysis of model performance using formula-only descriptors.** Classification accuracy and regression R^2^ values obtained using only composition-based descriptors, corresponding to Type *a* features in **Table 1**.

| **ML**  **Model** | **Classification** | | **Regression** | |
| --- | --- | --- | --- | --- |
|  | **Training**  **(Accuracy)** | **Test**  **(Accuracy)** | **Training**  **(R^2^)** | **Test**  **(R^2^)** |
| LASSO | - | - | 0.4193 | 0.4175 |
| DT | 0.7451 | 0.6957 | 0.5771 | 0.5379 |
| RF | 0.902 | 0.8261 | 0.4865 | 0.4319 |
| XGBoost | 0.8431 | 0.7391 | 0.8973 | 0.6593 |
| ANN | 0.6731 | 0.5909 | 0.6473 | 0.6077 |
| SVM | 0.8039 | 0.6957 | 0.8004 | 0.7119 |

References

[1].	Y. Zhang, T. T. Zuo, Z. Tang, M. C. Gao, K. A. Dahmen, P. K. Liaw and Z. P. Lu, “Microstructures and properties of high-entropy alloys,” *Progress in Materials Science* 61 (2014): 1-93, <https://doi.org/10.1016/j.pmatsci.2013.10.001>

[2].	B. J. Palmer, “Transverse-current autocorrelation-function calculations of the shear viscosity for molecular liquids,” *Physical Review E* 49 (1994): 359-366, <https://doi.org/10.1103/PhysRevE.49.359>

[3].	Z. K. Tang, J. S. Tse and L. M. Liu, “Unusual Li-Ion Transfer Mechanism in Liquid Electrolytes: A First-Principles Study,” *The Journal of Physical Chemistry Letters* 7 (2016): 4795-4801, <https://doi.org/10.1021/acs.jpclett.6b02351>

[4].	S. C. Kim, S. T. Oyakhire, C. Athanitis, J. Wang, Z. Zhang, W. Zhang, D. T. Boyle, M. S. Kim, Z. Yu, X. Gao, T. Sogade, E. Wu, J. Qin, Z. Bao, S. F. Bent and Y. Cui, “Data-driven electrolyte design for lithium metal anodes,” *Proceedings of the National Academy of Sciences* 120 (2023): e2214357120, <https://doi.org/10.1073/pnas.2214357120>

[5].	Y. Maeyoshi, D. Ding, M. Kubota, H. Ueda, K. Abe, K. Kanamura and H. Abe, “Long-Term Stable Lithium Metal Anode in Highly Concentrated Sulfolane-Based Electrolytes with Ultrafine Porous Polyimide Separator,” *ACS Applied Materials & Interfaces* 11 (2019): 25833-25843, <https://doi.org/10.1021/acsami.9b05257>

[6].	L. Suo, W. Xue, M. Gobet, S. G. Greenbaum, C. Wang, Y. Chen, W. Yang, Y. Li and J. Li, “Fluorine-donating electrolytes enable highly reversible 5-V-class Li metal batteries,” *Proceedings of the National Academy of Sciences* 115 (2018): 1156-1161, <https://doi.org/10.1073/pnas.1712895115>

[7].	X. Fan, L. Chen, X. Ji, T. Deng, S. Hou, J. Chen, J. Zheng, F. Wang, J. Jiang, K. Xu and C. Wang, “Highly Fluorinated Interphases Enable High-Voltage Li-Metal Batteries,” *Chem* 4 (2018): 174-185, <https://doi.org/10.1016/j.chempr.2017.10.017>

[8].	J.-D. Xie, J. Patra, P. Chandra Rath, W.-J. Liu, C.-Y. Su, S.-W. Lee, C.-J. Tseng, Y. A. Gandomi and J.-K. Chang, “Highly concentrated carbonate electrolyte for Li-ion batteries with lithium metal and graphite anodes,” *Journal of Power Sources* 450 (2020): 227657, <https://doi.org/10.1016/j.jpowsour.2019.227657>

[9].	S. Zhu and J. Chen, “Dual strategy with Li-ion solvation and solid electrolyte interphase for high Coulombic efficiency of lithium metal anode,” *Energy Storage Materials* 44 (2022): 48-56, <https://doi.org/10.1016/j.ensm.2021.10.007>
